# Supplementary material for: The Mechanism Underlying the Hypoglycemic Effect of Epimedin C on Mice with Type 2 Diabetes Mellitus Based on Proteomic Analysis
Source: Nutrients. 2023 Dec 21;16(1):25. doi: 10.3390/nu16010025 (PMC10780735; doi:10.3390/nu16010025)
Supplement: Supplementary file 1 [file nutrients-16-00025-s001.zip › nutrients-2754407-supplementary.pdf]

SUPPLEMENTARY MATERIALS

TABLES

Table S1. Experimental groups and treatments (*n* =10 in each group).

Group	Treatment	Intraperitoneal injection	Diet
NC (normal control) group	sterile distilled water	saline	control diet
MC (model control) group	sterile distilled water	streptozotocin	high-fat diet
PC1 (the first positive control) group	200 $mg \cdot kg^{-1}$ metformin	streptozotocin	high-fat diet
PC2 (the second positive control) group	5 $mg \cdot kg^{-1}$ glimepiride	streptozotocin	high-fat diet
EC30 (30 $mg \cdot kg^{-1}$ epimedin C) group	30 $mg \cdot kg^{-1}$ epimedin C	streptozotocin	high-fat diet
EC10 (10 $mg \cdot kg^{-1}$ epimedin C) group	10 $mg \cdot kg^{-1}$ epimedin C	streptozotocin	high-fat diet
EC5 (5 $mg \cdot kg^{-1}$ epimedin C) group	5 $mg \cdot kg^{-1}$ epimedin C	streptozotocin	high-fat diet

Table S2. Effects of epimedin C on fasting blood glucose (FBG) at 0 and 28 d.

**Group**	**FBG levels at 0 d (mmol·L^{-1})**	**FBG levels at 28 d (mmol·L^{-1})**	**Changes of FBG levels**	**Significant differences**
NC group	6.8±0.1 ^{d}	6.9±0.3 ^{d}	increase 1.47%	
MC group	18.7±0.5 ^{c}	21.5±1.1 ^{a}	increase 14.97%	*
PC1 group	22.6±0.6 ^{a}	14.5±0.7 ^{c}	decrease 35.84%	*
PC2 group	22.8±0.3 ^{a}	14.2±1.2 ^{c}	decrease 37.72%	*
EC30 group	18.7±1.6 ^{c}	13.5±1.1 ^{c}	decrease 27.81%	*
EC10 group	17.1±1.1 ^{b}	12.1±0.2 ^{b}	decrease 17.14%	*
EC5 group	21.0±0.3 ^{c}	17.4±1.0 ^{b}	decrease 6.86%	

Note: Different superscript letters indicate significant differences between groups (P<0.05); *, significant differences of FBG levels between 0 and 28 d (P<0.05).

Table S3. Effects of epimedin C on insulin contents in serums and homeostasis model assessment of insulin resistance (HOMA-IR).

**Group**	**Insulin content (mIU·L^{-1})**	**HOMA-IR**
NC group	9.6±0.7 ^{a}	2.9±0.5 ^{d}
MC group	7.6±0.3 ^{d}	7.0±1.0 ^{a}
PC1 group	9.0±0.1 ^{abc}	5.7±0.9 ^{abc}
PC2 group	9.2±0.4 ^{ab}	6.3±1.0 ^{ab}
EC30 group	8.9±0.7 ^{abc}	5.1±0.9 ^{bc}
EC10 group	8.1±0.2 ^{cd}	6.0±0.8 ^{ab}
EC5 group	7.8±0.7 ^{d}	5.5±1.1 ^{abc}

Note: Different superscript letters indicate significant differences between various groups (P<0.05).

Table S4. Gene ontology (GO) assay of key differentially expressed proteins (DEPs).

GO identity	Function	Protein/Gene	GO classification
GO:0044282	small molecule catabolic process	Acacb/ Dbi/ Thnsl2/ Acox2/ Got1/ Mat1a/ Oat/ Pck1/ Sult1b1/ Sult1e1	biological process
GO:0006631	fatty acid metabolic process	Acacb/ Cyp4a14/ Dbi/ Fads1/ Fads2/ Thnsl2/ Acox2/ Aldh3a2/ Cyp2a4/ Pck1/ Ptges3	biological process
GO:1901605	alpha-amino acid metabolic process	Gart/ Gm4952/ Thnsl2/ Adss/ Dglucy/ Got1/ Mat1a/ Oat	biological process
GO:0046394	carboxylic acid biosynthetic process	Acacb/ Cyp4a14/ Fads1/ Fads2/ Gart/ Thnsl2/ Got1/ Ptges3/ Rbp1	biological process
GO:0016053	organic acid biosynthetic process	Acacb/ Cyp4a14/ Fads1/ Fads2/ Gart/ Thnsl2/ Got1/ Ptges3/ Rbp1	biological process
GO:0016054	organic acid catabolic process	Acacb/ Dbi/ Thnsl2/ Acox2/ Got1/ Mat1a/ Oat/ Pck1	biological process
GO:0046395	carboxylic acid catabolic process	Acacb/ Dbi/ Thnsl2/ Acox2/ Got1/ Mat1a/ Oat/ Pck1	biological process
GO:0009156	ribonucleoside monophosphate biosynthetic process	Gart/ Prps1/ Umps/ Adss	biological process
GO:0016042	lipid catabolic process	Acacb/ Apob/ Ces3a/ Ces3b/ Dbi/ Pla2g12b/ Acox2/ Pck1/ Sult1e1	biological process
GO:0006520	cellular amino acid metabolic process	Gart/ Gm4952/ Thnsl2/ Adss/ Dglucy/ Got1/ Mat1a/ Oat	biological process
GO:0005852	eukaryotic translation initiation factor 3 complex	Abce1/ Eif3d/ Eif3g	cellular component
GO:0032994	protein-lipid complex	Apob/ Dbi/ Clu	cellular component
GO:0005767	secondary lysosome	Fth1/ Hrg	cellular component
GO:0005697	telomerase holoenzyme complex	Dkc1/ Ptges3	cellular component
GO:0030904	retromer complex	Snx2/ Snx5	cellular component
GO:0150034	distal axon	Ap1s1/ Ctsz/ Palld/ Clu/ Got1/ Myh10	cellular component
GO:0045178	basal part of cell	Ceacam1/ Eppk1/ Slco1a4	cellular component
GO:0034364	high-density lipoprotein particle	Apob/ Clu	cellular component
GO:0097038	perinuclear endoplasmic reticulum	Dbi/ Clu	cellular component
GO:0005732	small nucleolar ribonucleoprotein complex	Gar1/ Dkc1	cellular component
GO:0031406	carboxylic acid binding	Acacb/ Cyp4a14/ Thnsl2/ Acox2/	molecular function

		Got1/ Mat1a/ Pck1	
GO:0043177	organic acid binding	Acacb/ Cyp4a14/ Thnsl2/ Acox2/ Got1/ Mat1a/ Pck1	molecular function
GO:0016903	oxidoreductase activity, acting on the aldehyde or oxo group of donors	Aldh1a7/ Aox3/ Aldh3a2/ Htatip2	molecular function
GO:0019842	vitamin binding	Acacb/ Thnsl2/ Got1/ Oat/ Rbp1	molecular function
GO:0016831	carboxy-lyase activity	Umps/ Got1/ Pck1	molecular function
GO:1901567	fatty acid derivative binding	Acbd5/ Cyp4a14/ Dbi	molecular function
GO:0043531	ADP binding	Prps1/ Mat1a/ Myh10	molecular function
GO:1901681	sulfur compound binding	Acacb/ Acbd5/ Apob/ Dbi/ Prelp/ Hrg	molecular function
GO:0003743	translation initiation factor activity	Eif3d/ Eif3g/ Eif4e	molecular function
GO:0016829	lyase activity	Thnsl2/ Umps/ Dglucy/ Got1/ Pck1	molecular function

FIGURE LEGENDS

Figure S1. Chemical structure of epimedin C.

Glc, glucose; Rha, rhamnose.

Figure S2. Volcano plots of differentially expressed proteins (DEPs).

A, DEPs in NC vs MC groups; B, DEPs in EC30 vs MC groups; C, DEPs in NC vs EC30 groups.

Figure S3. Hierarchical clustering analysis of DEPs.

A, DEPs in NC vs MC groups; B, DEPs in EC30 vs MC groups; C, DEPs in NC vs EC30 groups.

Figure S4. Gene-concept network (CNET) assay of gene ontology (GO) functions.

A, biological process (BP) and key enriched DEPs; B, cellular component (CC) and key enriched DEPs; C, molecular function (MF) and key enriched DEPs.

Figure S5. Typical Kyoto encyclopedia of genes and genomes (KEGG) pathways.

A, peroxisome proliferator-activated receptor (PPAR) signaling pathway; B, glycolysis/gluconeogenesis.

Red boxes represent up-regulated DEPs, and blue boxes represent down-regulated DEPs.

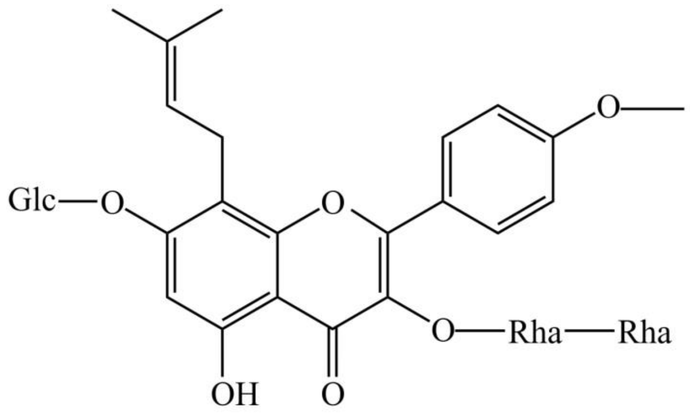

Figure S1

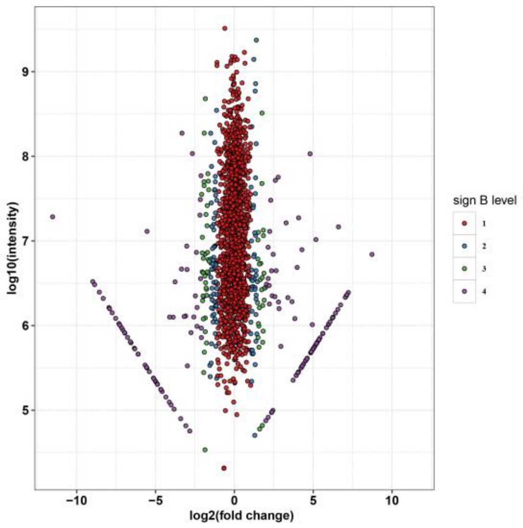

A

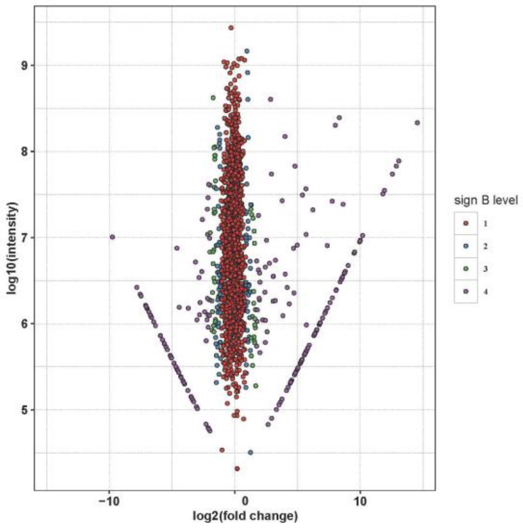

B

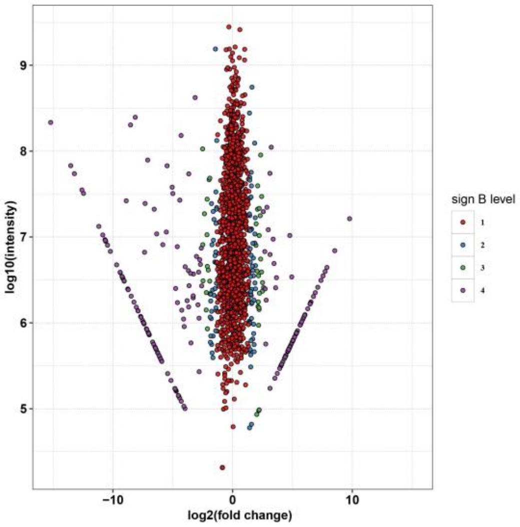

C

Figure S2

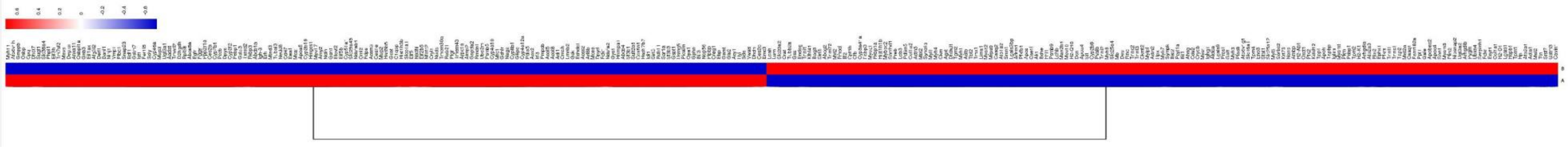

A

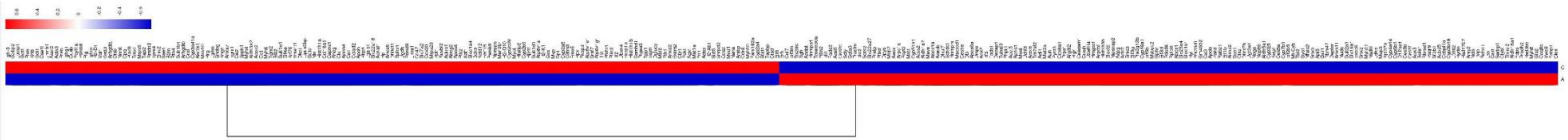

B

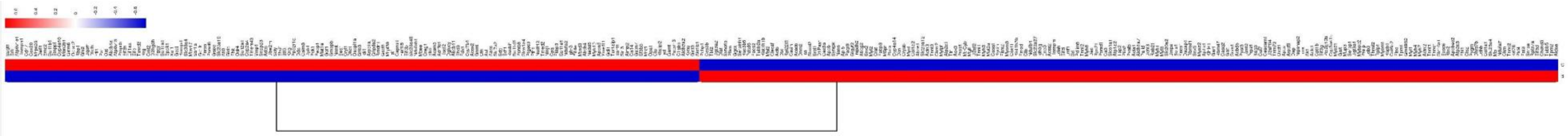

C

Figure S3

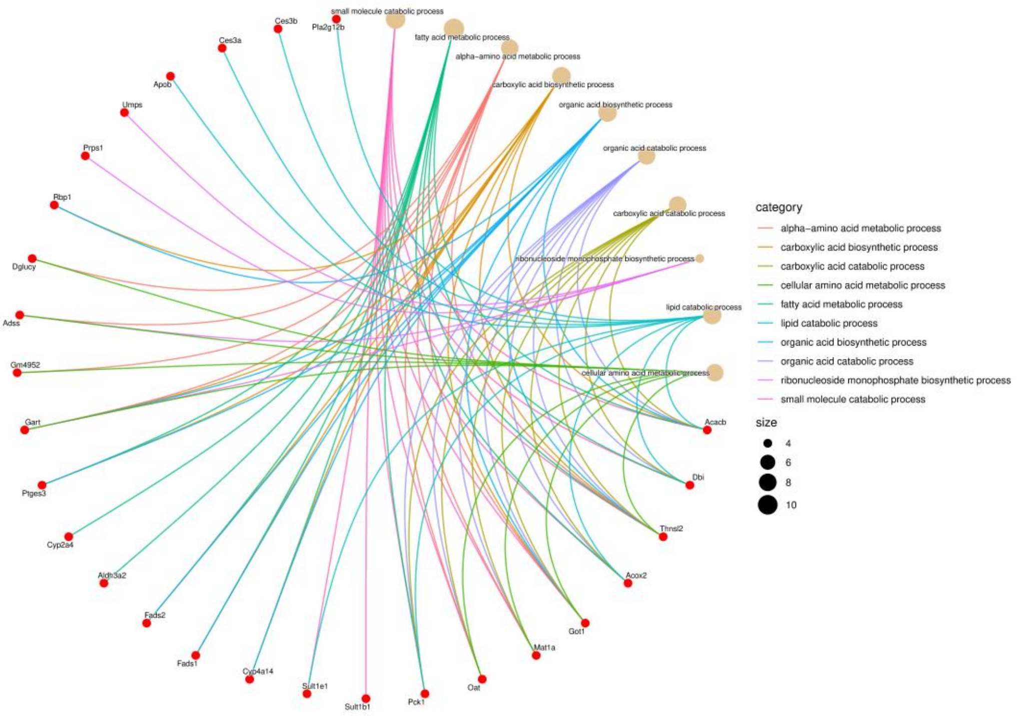
small molecule catabolic process
fatty acid metabolic process
alpha−amino acid metabolic process
carboxylic acid biosynthetic process
organic acid biosynthetic process
organic acid catabolic process
carboxylic acid catabolic process
ribonucleoside monophosphate biosynthetic process
lipid catabolic process
cellular amino acid metabolic process
Ces3b
Pla2g12b
Ces3a
Apob
Umps
Prps1
Rbp1
Dglucy
Adss
Gm4952
Gart
Ptges3
Cyp2a4
Aldh3a2
Fads2
Fads1
Cyp4a14
Sult1e1
Sult1b1
Pck1
Oat
Mat1a
Got1
Acox2
Thnsl2
Dbi
Acacb
category
alpha−amino acid metabolic process
carboxylic acid biosynthetic process
carboxylic acid catabolic process
cellular amino acid metabolic process
fatty acid metabolic process
lipid catabolic process
organic acid biosynthetic process
organic acid catabolic process
ribonucleoside monophosphate biosynthetic process
small molecule catabolic process
size
4
6
8
10

A

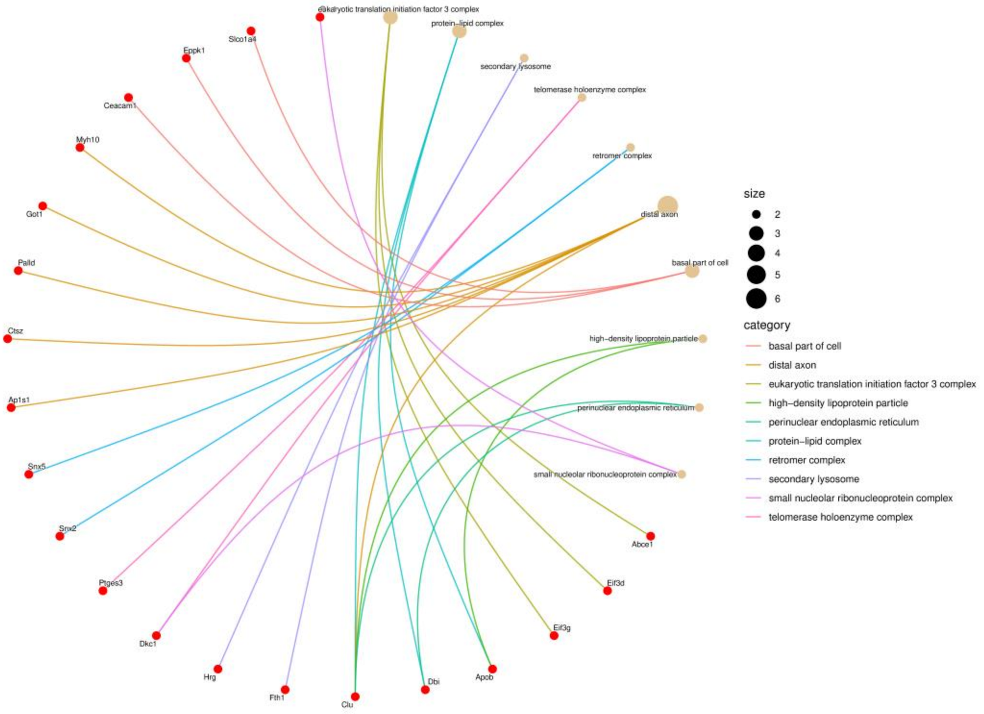
eukaryotic translation initiation factor 3 complex
protein−lipid complex
secondary lysosome
telomerase holoenzyme complex
retromer complex
distal axon
basal part of cell
high−density lipoprotein particle
perinuclear endoplasmic reticulum
small nucleolar ribonucleoprotein complex
Slco1a4
Eppk1
Ceacam1
Myh10
Got1
Palld
Ctsz
Ap1s1
Snx5
Snx2
Ptges3
Dkc1
Hrg
Fth1
Clu
Dbi
Apob
Eif3g
Eif3d
Abce1
size
2
3
4
5
6
category
basal part of cell
distal axon
eukaryotic translation initiation factor 3 complex
high−density lipoprotein particle
perinuclear endoplasmic reticulum
protein−lipid complex
retromer complex
secondary lysosome
small nucleolar ribonucleoprotein complex
telomerase holoenzyme complex

B

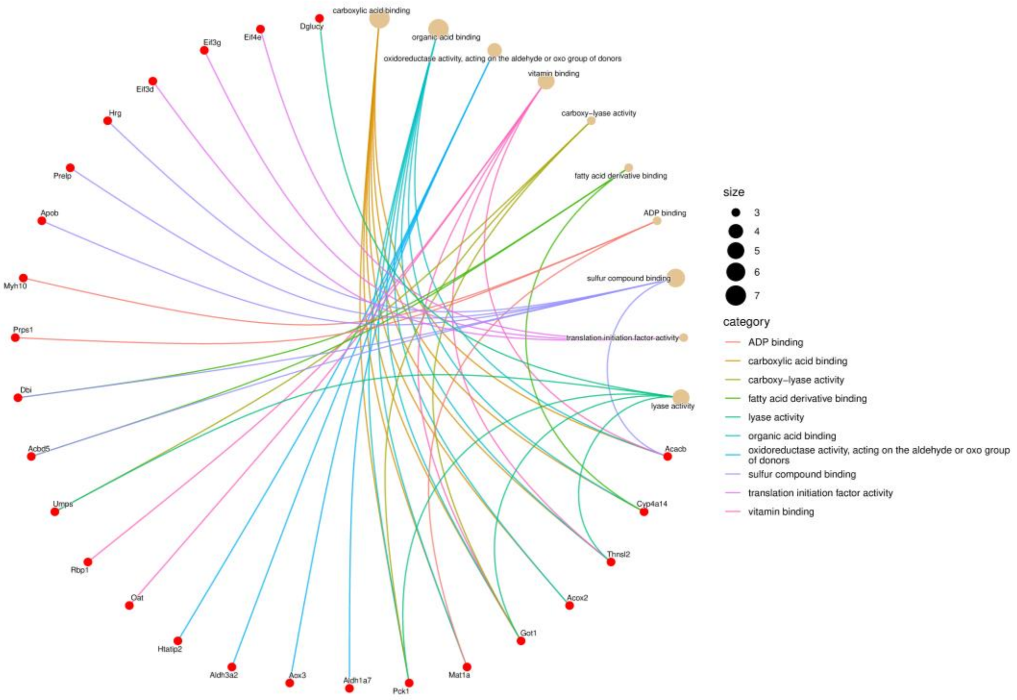
carboxylic acid binding
organic acid binding
oxidoreductase activity, acting on the aldehyde or oxo group of donors
vitamin binding
carboxy−lyase activity
fatty acid derivative binding
ADP binding
sulfur compound binding
translation initiation factor activity
lyase activity
Dglucy
Eif4e
Eif3g
Eif3d
Hrg
Prelp
Apob
Myh10
Prps1
Dbi
Acbd5
Umps
Rbp1
Oat
Htatip2
Aldh3a2
Aox3
Aldh1a7
Pck1
Mat1a
Got1
Acox2
Thnsl2
Cyp4a14
Acacb
size
3
4
5
6
7
category
ADP binding
carboxylic acid binding
carboxy−lyase activity
fatty acid derivative binding
lyase activity
organic acid binding
oxidoreductase activity, acting on the aldehyde or oxo group of donors
sulfur compound binding
translation initiation factor activity
vitamin binding

C

Figure S4

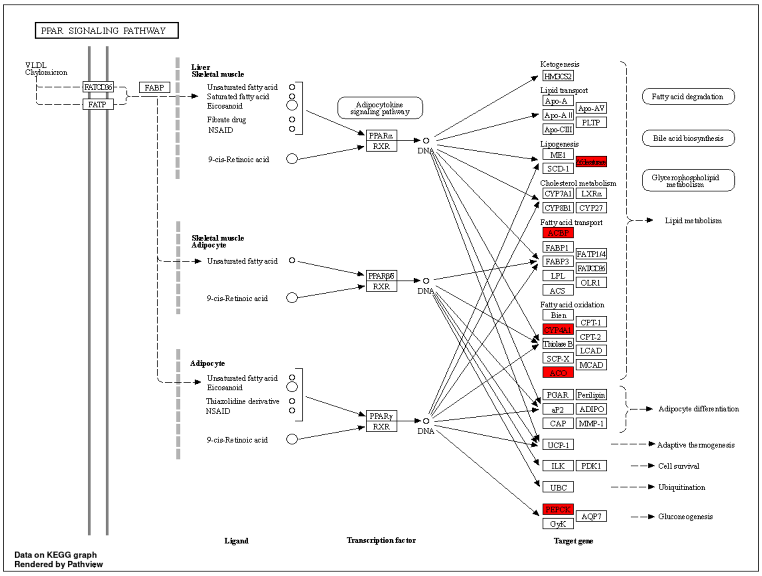
PPAR SIGNALING PATHWAY
VLDL
Chylomicron
FATCD36
FATP
FABP
Liver
Skeletal muscle
Unsaturated fatty acid
Saturated fatty acid
Eicosanoid
Fibrate drug
NSAID
9-cis-Retinoic acid
Adipocytokine signaling pathway
PPARα
RXR
DNA
Skeletal muscle
Adipocyte
Unsaturated fatty acid
9-cis-Retinoic acid
PPARβ/δ
RXR
DNA
Adipocyte
Unsaturated fatty acid
Eicosanoid
Thiazolidine derivative
NSAID
9-cis-Retinoic acid
PPARγ
RXR
DNA
Ketogenesis
HMGCS2
Lipid transport
Apo-A
Apo-AV
Apo-A II
PLTP
Apo-CIII
Lipogenesis
ME1
Δ6desaturase
SCD-1
Cholesterol metabolism
CYP7A1
LXRα
CYP8B1
CYP27
Fatty acid transport
ACBP
FABP1
FATP1/4
FABP3
FATCD36
LPL
OLR1
ACS
Fatty acid oxidation
Bien
CPT-1
CYP4A1
CPT-2
Thiolase B
LCAD
SCP-X
MCAD
ACO
PGAR
Perilipin
aP2
ADIPO
CAP
MMP-1
UCP-1
ILK
PDK1
UBC
PEPCK
AQP7
GyK
Fatty acid degradation
Bile acid biosynthesis
Glycerophospholipid metabolism
Lipid metabolism
Adipocyte differentiation
Adaptive thermogenesis
Cell survival
Ubiquitination
Gluconeogenesis
Ligand
Transcription factor
Target gene
Data on KEGG graph
Rendered by Pathview

Figure S5
